# Combination protein biomarkers predict multiple sclerosis diagnosis and outcomes

**DOI:** 10.1186/s12974-024-03036-4

**Published:** 2024-02-17

**Authors:** Eleftheria Kodosaki, W. John Watkins, Sam Loveless, Karim L. Kreft, Aidan Richards, Valerie Anderson, Lisa Hurler, Neil P. Robertson, Wioleta M. Zelek, Emma C. Tallantyre

**Affiliations:** 1 UK Dementia Research Institute at University College London, London, WC1E6BT UK; 2 Department of Neurodegenerative Disease, UCL Institute of Neurology, Queen Square, London, WC1N3BG UK; 3 Division of Psychological Medicine and Clinical Neurosciences, School of Medicine, Cardiff University, Cardiff, CF14 4XW UK; 4 Division of Infection and Immunity, School of Medicine, Cardiff University, Cardiff, UK; 5 Department of Neurology, University Hospital of Wales, Cardiff, UK; 6 Department of Internal Medicine and Haematology, Semmelweis University, Budapest, 1085 Hungary

**Keywords:** Biomarkers, Multiple sclerosis, Cerebrospinal fluid, Serum, Complement, Prediction

## Abstract

Establishing biomarkers to predict multiple sclerosis diagnosis and prognosis has been challenging using a single biomarker approach. We hypothesised that a combination of biomarkers would increase the accuracy of prediction models to differentiate multiple sclerosis from other neurological disorders and enhance prognostication for people with multiple sclerosis. We measured 24 fluid biomarkers in the blood and cerebrospinal fluid of 77 people with multiple sclerosis and 80 people with other neurological disorders, using ELISA or Single Molecule Array assays. Primary outcomes were multiple sclerosis versus any other diagnosis, time to first relapse, and time to disability milestone (Expanded Disability Status Scale 6), adjusted for age and sex. Multivariate prediction models were calculated using the area under the curve value for diagnostic prediction, and concordance statistics (the percentage of each pair of events that are correctly ordered in time for each of the Cox regression models) for prognostic predictions. Predictions using combinations of biomarkers were considerably better than single biomarker predictions. The combination of cerebrospinal fluid [chitinase-3-like-1 + TNF-receptor-1 + CD27] and serum [osteopontin + MCP-1] had an area under the curve of 0.97 for diagnosis of multiple sclerosis, compared to the best discriminative single marker in blood (osteopontin: area under the curve 0.84) and in cerebrospinal fluid (chitinase-3-like-1 area under the curve 0.84). Prediction for time to next relapse was optimal with a combination of cerebrospinal fluid[vitamin D binding protein + Factor I + C1inhibitor] + serum[Factor B + Interleukin-4 + C1inhibitor] (concordance 0.80), and time to Expanded Disability Status Scale 6 with cerebrospinal fluid [C9 + Neurofilament-light] + serum[chitinase-3-like-1 + CCL27 + vitamin D binding protein + C1inhibitor] (concordance 0.98). A combination of fluid biomarkers has a higher accuracy to differentiate multiple sclerosis from other neurological disorders and significantly improved the prediction of the development of sustained disability in multiple sclerosis. Serum models rivalled those of cerebrospinal fluid, holding promise for a non-invasive approach. The utility of our biomarker models can only be established by robust validation in different and varied cohorts.

**Supplementary Information:**

The online version contains supplementary material available at 10.1186/s12974-024-03036-4.

## Introduction

Multiple sclerosis is one of the most common causes of neurological disability in young adults and long-term disease outcomes are highly variable and difficult to predict at onset. Since the emergence of disease modifying therapies for multiple sclerosis in the 1990s, more than 15 treatments with variable efficacy are now available. However, uncertainty remains over selection and sequencing of disease modifying therapies; aggressive suppression of early multiple sclerosis activity may improve longer-term disease outcomes, but most high-efficacy disease modifying therapies carry risks of serious adverse events [1, 2]. Therefore, there is an urgent need to identify multiple sclerosis biomarkers than can inform disease prognosis, treatment response and potential for adverse events, to enable a more personalised approach to therapeutic interventions.

The hallmark of multiple sclerosis pathology is inflammatory demyelination within the white and grey matter of the brain and spinal cord. Foci of acute inflammation and demyelination are thought to explain the short-lived flare-ups of symptoms (relapses), seen early in multiple sclerosis, but a more complex interplay between immune cells, glia and neurons is likely to explain accumulation of longer-term and/or progressive disability [3, 4]. Recent studies supporting the use of the neuronal protein neurofilament light as a prognostic multiple sclerosis biomarker have offered some optimism for personalising treatment decisions. The prognostic value of neurofilament light in multiple sclerosis is well-established when measured in CSF using ELISA-based assays [5], but this represents an impractical tissue to sample repeatedly. Serum neurofilament light concentrations are an order of magnitude lower and poorly detected using ELISA but can now be accurately measured using single molecule array (SiMoA) assays [6]. Good correlation is observed between serum and CSF concentrations of neurofilament light, a feature which has also been demonstrated for some other candidate multiple sclerosis biomarkers. As a result serum/plasma analysis of selected biomarkers suggests promise for a less invasive approach to disease monitoring [7, 8]. However, group effects observed in neurofilament light studies appear to translate to relatively modest individual predictions, especially when using a single cross-sectional measurement, raising questions over its clinical utility [9]. Other proteins including glial marker chitinase-3-like-1 [10], and inflammatory cytokine osteopontin [11], have also been identified as potential candidates for predicting multiple sclerosis outcomes, and may offer complementary information to neurofilament light. Candidate multiple sclerosis biomarkers are often studied in isolation, and comparing studies that have used different clinical outcomes is challenging [12]. Given the complexity of multiple sclerosis biology, a combination of markers may improve predictions versus any marker in isolation.

In this study, we investigate 31 candidate biomarkers for their ability to distinguish people with multiple sclerosis from people with other neurological conditions, and predict short- and medium-term clinical outcomes in those with multiple sclerosis. We use paired CSF and serum samples from 157 people to study the optimal combination of markers for diagnostic and prognostic predictions.

## Materials and methods

We identified candidate biomarkers during a literature search (conducted in 2018, using PubMed database and search terms “multiple sclerosis” and “biomarker” ± “CSF” or “serum” or “plasma”). We identified candidate tissue biomarkers which had been shown to differentiate between multiple sclerosis and controls, between multiple sclerosis subtypes, or to predict severity of the disease (Table 1) [12]. Candidate biomarkers included pro- and anti-inflammatory cytokines (e.g. IL-6, IL-18, and IL-10), proteins and factors involved in the CNS (such as brain-derived neurotrophic factor (BDNF), osteopontin and glial fibrillary acidic protein (GFAP)), chemokines (such as CXCL12 and CXCL13), complement proteins and proteins that are involved in other pathways relevant to multiple sclerosis (such as Vitamin D binding protein).

**Table 1:** Description of candidate biomarkers studied in this cohort

Marker	Description	Evidence for relevance in multiple sclerosis
CXCL12	Chemokine/cytokine-related	Increased in CSF of people with PPMS vs RRMS [48], increased in people with multiple sclerosis vs controls [49]
CXCL13	Chemokine/cytokine-related	Increased in CSF of people with multiple sclerosis and correlated with relapse rate [50]
IL-6	Chemokine/cytokine-related	Increased in CSF of people with multiple sclerosis, and correlated with disease activity in RRMS [51]
IL-10	Chemokine/cytokine-related	Decreased in people with CIS who relapsed [52]. In multiple sclerosis, low IL-10 was associated with higher disability [53], and high IL10 with inactive disease [54]
IL-12/IL-23p40	Chemokine/cytokine-related	Found upregulated in multiple sclerosis serum [55] and is higher in people with RRMS vs controls [54]
Soluble CD27	Chemokine/cytokine-related	Increased levels of the soluble CD27 are found in CSF of people with multiple sclerosis, and there is a correlation between IgG index and CD27[32]
CRP	Inflammatory marker	Serum CRP/albumin ratio are correlated with higher multiple sclerosis disease activity and relapses [56], as well multiple sclerosis disease subtype[57], whereas serum CRP can predict depressive symptoms in newly diagnosed people with multiple sclerosis [58]
GFAP	CNS marker	CSF GFAP has been found to be higher in people with CIS/RRMS versus controls [59]. GFAP levels in serum and CSF have been linked with disease duration and severity, respectively [60]
CHi3L1	CNS marker	CSF chitinase-3-like-1 has been found to be predictive of conversion from CIS to multiple sclerosis [10]. CSF and serum chitinase-3-like-1 were both increased in more advanced multiple sclerosis [61]
Osteopontin	Chemokine/cytokine-related	Osteopontin levels in CSF have been found to be associated with disease activity [11] and disease progression [62], and were found increased in the CSF of people with multiple sclerosis vs controls [63]
TGF-β	CNS marker	CSF levels of TGF-β were higher in people with active multiple sclerosis [64], while lower TGF-β was found in the blood of people with multiple sclerosis when compared to controls [65]
MCP-1	Chemokine/cytokine-related	MCP-1 levels were decreased in the CSF of multiple sclerosis patients when compared to controls [29, 66]
BDNF	CNS marker	BDNF levels were decreased in the blood of people with multiple sclerosis vs controls, and lower in people with SP multiple sclerosis than RR multiple sclerosis[67]
IL-4	Chemokine/cytokine-related	Serum and CSF IL-4 was found to be increased in people with multiple sclerosis vs controls [68–70]
CCL27	Chemokine/cytokine-related	Serum CCL27 levels were higher in newly diagnosed and acute multiple sclerosis cases [30]
IFNγ	Chemokine/cytokine-related	In multiple sclerosis, blood levels of IFNγ were increased prior to the manifestation of symptoms [71]
IL-8	Chemokine/cytokine-related	CSF levels of IL-8 were found to be significantly higher in people with multiple sclerosis vs controls, but the serum levels were lower in people with multiple sclerosis vs controls [72]
TNFr1 (s)	Chemokine/cytokine-related	CSF levels of soluble TNFr1 were higher in people with multiple sclerosis vs controls, and were also associated with disease burden (lesion volume) [73]
TNFα	Chemokine/cytokine-related	Serum TNFα levels correlated with disease activity in RRMS [54] and was found higher in people with multiple sclerosis versus healthy controls [74]
IL-18	Chemokine/cytokine-related	Serum IL-18 was found to be higher in people with multiple sclerosis vs controls, higher in SPMS compared to RRMS, and higher during relapses [75]
Vitamin D binding protein	Metabolic marker	Serum from people with multiple sclerosis had high levels of DBP [76], however a different study found the opposite for newly diagnosed patients [37]
LIF	Chemokine/cytokine-related	CSF and serum LIF levels were higher in people with multiple sclerosis vs controls [77]
Complement proteins TCC, iC3b, C3, C5, C9, fH, fB, fI, C1inh/C1s complex	Immune system	Several complement system components and proteins have been found to be altered in CIS and multiple sclerosis, e.g. C3, C4, C4a, C1inhibitor and Factor H were found increased in the plasma of people with multiple sclerosis, whereas C9 was found decreased [78]. A study focusing on disease progression and response to treatment identified C4a and C3 as potential biomarkers of disease progression and subtype [79]. One study found C4b to be elevated in multiple sclerosis [80]
Neurofilament-light	CNS marker	Although less sensitive when measured in blood [81] several studies have found neurofilament light to be a predictive and diagnostic biomarker in multiple sclerosis (significantly higher in multiple sclerosis vs controls), higher in people with progressive vs RRMS, as well as in people with concurrent disease activity vs people in remission. Data are summarised in a recent systematic review and meta-analysis [82]

*SiMoA* single molecular array assays, *CSF* cerebrospinal fluid, *Ch3L1* chitinase-3-like-1, *IL (e.g. IL-6)* interleukin, *BDNF* brain derived neurotrophic factor, *CXCLx* chemokine (C-X-C motif) ligand x, *GFAP* glial fibrillary acidic protein, *pwMS* people with multiple sclerosis, *EDSS* Expanded Disability Status Scale, *TCC* terminal complement complex protein, *Cx (e.g. C9)* complement protein x, *C1inh/C1s* complex between C1 inhibitor and C1s proteins, *fx (e.g. fB)* complement factor x, *IFNγ* Interferon gamma, *TGFβ* transforming growth factor beta, *TNFα* tumour necrosis factor alpha, *BCA* bicinchoninic acid, *CDx (e.g. CD27)* cluster of differentiation protein x, *iC3b* inactivated C3b, *CRP* C-reactive protein, *MCP-1* monocyte chemoattractant protein 1, *IL-12/IL-23p40* the p40 subunit of the IL-12 family, *CCLx (e.g. CCL27)* C–C motif chemokine ligand x, *TNFr1 (s)* Soluble form of the TNFα receptor 1, *LIF* leukaemia inhibitory factor, *RRMS* relapsing–remitting multiple sclerosis, *S/PPMS* secondary/primary progressive multiple sclerosis

### Participants

People with multiple sclerosis were recruited as part of a long running observational study in South East Wales, UK (Ethics REC Ref: 05/ WSE03/111, 19/WA/0289), which has been described previously [13]. Briefly, participants are invited to annual clinic visits and data are collected at each clinical encounter, including measurement of expanded disability status scale (EDSS) [14], relapse history, disease subtype and disease modifying therapy review. People with new neurological symptoms lasting > 24 h in the absence of infection are reviewed by clinicians to confirm relapse (in the absence of an alternative explanation). Annual clinic data are complemented by a postal questionnaire, which includes an assessment of current disability using a validated self-reported EDSS tool [15]. Where possible, blood and CSF samples are acquired at the time of diagnostic lumbar puncture and archived in a biorepository. We studied paired serum and CSF samples from 77 people who were being investigated for demyelinating disease, all of whom ultimately reached a diagnosis of multiple sclerosis according to contemporary criteria, and 80 controls who were undergoing CSF examination for reasons other than suspected demyelinating disease; none of whom were diagnosed with multiple sclerosis during follow-up. Control samples were obtained from the Welsh Neuroscience Research Tissue Bank (WNRTB Ethics Rec Ref: 19/WA/0058). The demographic and clinical characteristics of the cohort are summarised in Table 2.

**Table 2:** Demographic and clinical characteristics of the cohort

	Non-multiple sclerosis (*n* = 80)	Multiple sclerosis (*n* = 77)
Age at sampling—years—mean (SD)	33.2 (10.2)	41.9 (12.4)
Gender—count (%)	Female 72 (90%) Male 8 (10%)	Female 46 (60%) Male 31 (40%)
Diagnosis—count	IIH 59 Primary headache 10 Sensory disturbance 3 FND 3 Epilepsy 2 SPS 1 Arthritis 1 Viral meningitis 1	RRMS 57 SPMS 10 PPMS 10
Baseline EDSS—median (range)		2.5 (1.0–7.5)
Follow-up duration—mean (SD)	–	7.9 (3.5) years
Disease modifying therapy (ever exposed)—count	–	Yes 46* (High efficacy 27) No 31
Relapse(s) experienced during follow-up—count (%)	–	46^+^ (60%)
Reached EDSS 6—count (%)	–	23 (30%)
Oligoclonal bands in CSF	–	Unpaired 53 (69%) Paired 9 (12%) Negative 15 (19%)

*IIH* idiopathic intracranial hypertension, *FND* functional neurological disorder, *SPS* stiff person syndrome, *RRMS* relapsing–remitting multiple sclerosis, *S/PPMS* secondary/primary progressive multiple sclerosis. High efficacy disease modifying therapy: monoclonal antibody or cladribine treatment; *Only 2 people with multiple sclerosis were exposed to disease modifying therapy at the time of sampling. ^+^ 36 experienced their relapse prior to commencing disease modifying therapy (prior to censor)

### Samples

Venous blood samples were collected in a BD vacutainer (Gold SST II Advance) between 04/2007 and 01/2020. Whole blood and paired CSF samples were immediately centrifuged at 4500 rpm for 10min at 4 °C within 2–3 h of collection. The resulting supernatant (serum or CSF) was subsequently split into 300 μl aliquots and stored at – 80 °C until use.

### Assays

In-house ELISA assays were used for the measurement of complement biomarkers (terminal complement complex (TCC), iC3b, C3, C5, C9, factor H, factor B, factor I) in serum and CSF. C1inhibitor/C1s complex (C1inh/C1s) was measured using a commercially available assay donated by Hycult Biotech (Cat No HK399). For all other biomarkers commercial assays from R&D were used (Additional file 1: Table S1). Dilutions of serum and CSF for all assays are described in Additional file 1: Table S1. For the complement assays, Nunc Maxisorp (VWR, UK) plates were coated with affinity-purified capture antibody overnight at 4 °C. Wells were subsequently washed 1 × with phosphate-buffered saline 0.1% Tween20 (Sigma Aldrich, Germany) (PBS-T) and blocked for 1 h at 37 °C with 1% bovine serum albumin (BSA) in PBS-T. After 1 wash, purified protein standards or serum samples (in duplicate), optimally diluted in 0.1% BSA in PBS-T, 5mM ethylenediaminetetraacetic acid (EDTA), were added to wells in duplicate and incubated for 1.5 h at 37 °C. Wells were washed 3 × with PBS-T then incubated for 1 h at 37 °C with detection antibody (either unlabelled or labelled in-house using horseradish peroxidase (HRP, where EZ-Link Plus Activated Peroxidase Kit (ThermoFisher Scientific, #31489, UK)) or biotin for the TCC assay (#21327) and washed 3 times with PBS-T. For unlabelled detection antibodies, HRP-labelled secondary antibody (anti-mouse or anti-rabbit IgG as appropriate (Jackson ImmunoResearch #715-035-151, #711-035-152, USA), or streptavidin-HRP (R&D systems, #DY995, UK) for TCC detection was added to wells, incubated and washed as above. Signals were detected using 3,3',5,5'-tetramethylbenzidine (TMB) (1-Step™ Ultra TMB-ELISA Substrate Solution, Thermofisher Scientific, UK) and after the addition of 2M sulfuric acid, absorbance (450nm) was measured. In each plate, protein standards were included, and samples were randomly assigned to eliminate assay bias. A nonlinear regression model was used to fit standard curves generated by ELISA. Biomarkers levels were automatically calculated by reference to the standard curve using GraphPad Prism. To account for any difference in the degree of blood–brain barrier permeability between people with multiple sclerosis and controls, the CSF total protein concentration was measured using micro-BCA Protein Assay Kit #23235, ThermoFisher Scientific, UK) [16], and CSF biomarker concentrations were adjusted accordingly. For the non-complement assays (Table 2 for details), manufacturer’s instructions were followed.

During optimisation, it was found that analytes were undetectable in the majority of CSF and serum samples using 5 assays (IFNγ, TGFβ, TNFα, IL-6, IL-12), so these were not taken forward into the main analysis. Analytes from six assays (GFAP, BDNF, IL-10, IL-18, LIF, CCL27, CXCL13) were undetectable in the CSF, and were thereafter used only in serum samples. One analyte (IL-8) was undetectable in serum, and thereafter only used in CSF. For all ELISA assays, intra-assay CVs were less than 10%, and inter-assay CVs were under 15%. For values outside the standard curve, the samples were re-measured using different dilutions. For neat samples, values below the standard curve were imputed (see Statistical analysis section and Additional file 1: Table S2).

Single molecule array (SiMoA) technology was employed to quantify neurofilament light in serum samples in view of the known insensitivity of ELISA for serum neurofilament light [6]. We used an HD-X analyser, a fully automated multiplex digital immunoassay instrument providing ultra-sensitive measurements (fg/ml) of proteins of biological interest, over a wide dynamic range and with low coefficients of variance. Serum samples were quantified using the human neurofilament light single bead-based advantage assay (Quanterix cat#103186), array discs (cat#103347) and assay buffers (cat#100488), following the manufacturers protocol.

### Statistical analysis

The demographic and clinical characteristics of the cohort were presented using descriptive statistics. Due to group differences between the multiple sclerosis and control cohorts, all analyses were corrected for age and sex. The concentration of each biomarker for both serum and CSF was individually compared between multiple sclerosis and controls using the non-parametric Mann–Whitney test. The Benjamini–Hochberg correction was applied to the p-values to control for the probability of type 1 error with repeated tests. Concentration of each biomarker (CSF and serum) were individually converted to *Z*-scores to allow all their model coefficients to be directly compared. *Z*-scores were generated by subtracting the mean and dividing by the standard deviation for all the valid data for each biomarker. To avoid zero values, levels for samples which were below the minimum detection limit were imputed using a value equal to half of the minimum detectable value (using our assays and samples). Due to scarce CSF or serum for some patients, it was not possible to measure all analytes in all samples. The frequency of missing and imputed data is shown in Additional file 1: Table S2, but did not differ significantly between MS and control cases.

The *Z*-scores were used to model the utility of single and combination biomarkers of predicting disease status and clinical outcome(s). For determination of MS versus control status, a cross-validation approach was taken to avoid over-fitting of a model. The cohort was randomly divided into four roughly equally sized and distinct groups (Additional file 1: Table S3 and S4). Three of these four groups were separately used as “training datasets” to produce a model using roughly 75% of the data, which was then tested on the remaining approximately 25% of the data (the “test dataset”). This was repeated 4 times with each of the 4 groups being the test dataset with the other 3 producing the model. In investigating the value of biomarkers to distinguish multiple sclerosis from control categories, receiver operating characteristic curve (ROC) curves were produced and the area under the curve (AUC) was used to calculate the ability of single biomarkers (CSF and serum) and combinations of biomarkers, to distinguish multiple sclerosis from control cases. After looping through all combinations of biomarkers, a mean test AUC was calculated from the AUCs from the 4 test cohorts. Mean AUC values were ordered from lowest to highest, and the optimum model was selected when addition of a further analyte resulted in an AUC increase < 0.01 (worked example provided in Additional file 1). CSF and serum biomarkers were explored independently in models adjusted for age and sex, and finally data on both serum and CSF markers were combined in a single adjusted model. To explore the reliability of the results, we re-ran the models 1000 times on random selections of Train and Test data, to generate data on the range of AUCs produced.

We performed a sensitivity analysis to explore the possible association between biomarker concentrations with age and sex. We built a linear model of biomarkers levels adjusted for age and sex as predictors in the control cohort. We used this to generate predicted values for each biomarker and subtracted those from the measured biomarker concentrations in both control and MS cohorts, to adjust for any physiological effect of age and sex on biomarker concentration, so that only the MS effects of the biomarkers would be reflected by the model. Using the ‘corrected’ biomarker concentrations, we repeated the modelling as above.

To investigate the prognostic value of single and combinations of biomarkers, we used Cox regression models. We used two different dependent variables (outcomes) to reflect different aspects of multiple sclerosis biology. Firstly, we studied time from sampling (biomarker measurement) to next relapse for all people with multiple sclerosis. To mitigate the effect of disease modifying therapy on suppressing relapses, we censored follow-up at the time of disease modifying therapy commencement. Secondly, we studied time to EDSS 6 (need for unilateral assistance to walk 100m), sustained for at least 6 months, adjusted according to whether people had ever versus never (1 versus 0) received disease modifying therapy during follow-up. Only EDSS scores measured > 30 days out of relapse were used, to account for temporary fluctuations in disability that are likely to recover. The concordance statistic, i.e. the percentage of each pair of events that are correctly ordered in time for each of the Cox regression models, was used to rank best fit. After looping through all combinations of biomarkers, a mean concordance statistic was calculated from the concordance statistics from the 4 test cohorts. Mean concordance values were ordered from lowest to highest, and the optimum model was selected when addition of a further analyte resulted in a concordance increase < 0.01.

All statistical analysis was performed using R version 4.2.1 (2022-06-23 ucrt), R packages pROC (1.18.0), survival (3.3-1), survminer (0.4.9), and SPSS 27.

## Results

### CSF and serum biomarkers in people with multiple sclerosis versus controls

**Fig. 1:**
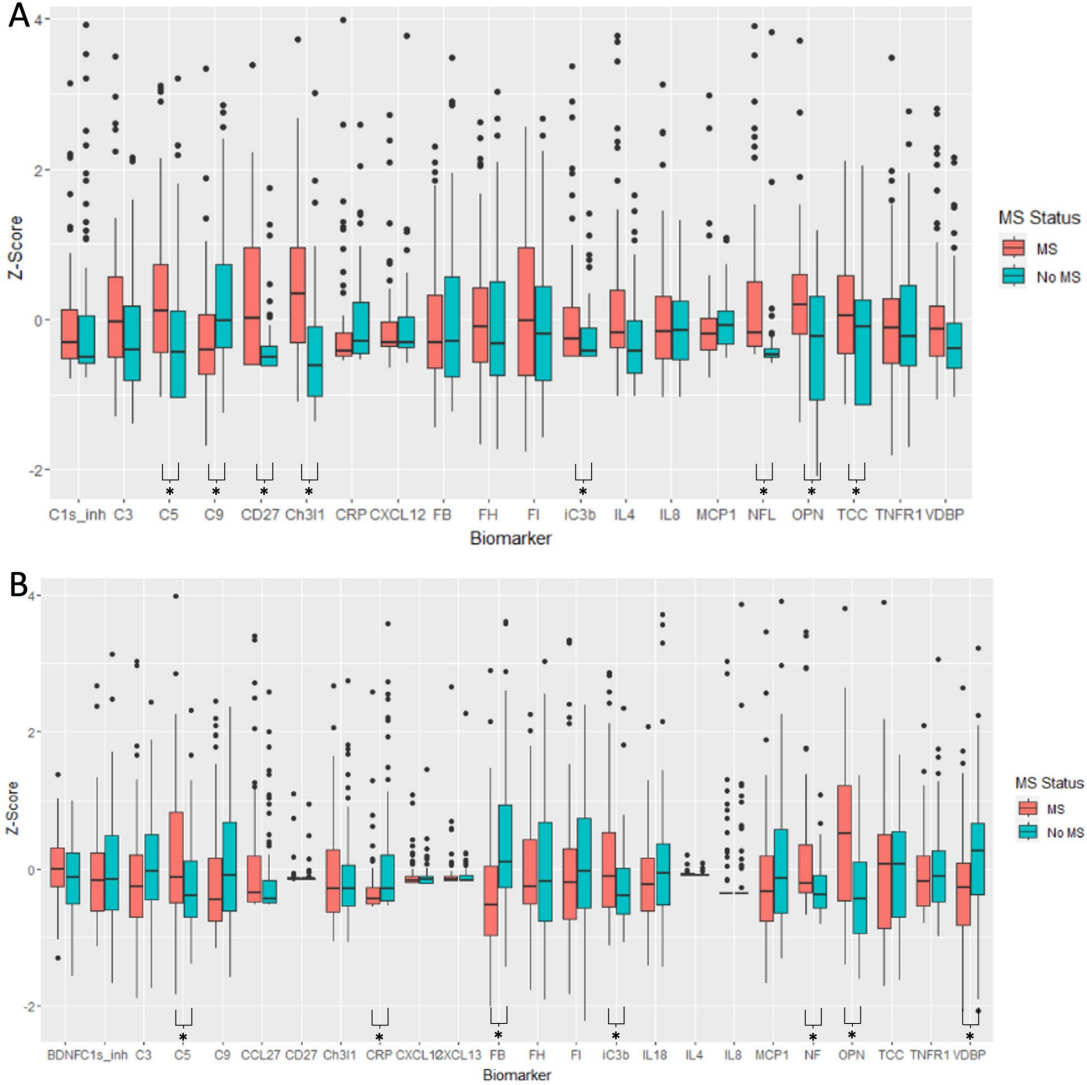
Concentrations of CSF and serum biomarkers in multiple sclerosis versus controls. **A** CSF and **B** serum samples, values expressed as *Z*-scores

Eight markers were found to be significantly differently expressed in CSF of multiple sclerosis versus control cases: chitinase-3-like-1, soluble CD27 (sCD27), neurofilament light, osteopontin, C5, iC3b, C9 and TCC (Additional file 1: Table S4). Those with the highest fold difference in CSF between multiple sclerosis and controls were sCD27 (4.1-fold increase in multiple sclerosis versus controls), neurofilament light (3.4-fold increase in multiple sclerosis) and iC3b (2.6-fold increase in multiple sclerosis). Seven markers were found to be significantly differently expressed in the serum of multiple sclerosis versus controls: osteopontin, Factor B, vitamin D binding protein, C5, iC3b, CRP and neurofilament light (Fig. 1, Additional file 1: Table S5). Neurofilament-light (1.6-fold increase in multiple sclerosis) and osteopontin (1.54-fold increase in multiple sclerosis) were the serum biomarkers with the highest fold difference.

### Statistical models of CSF and serum analytes differentiate multiple sclerosis from controls

The AUC for predicting multiple sclerosis versus control status using age and sex alone was 0.77. sCD27 and neurofilament light were the single CSF biomarkers that produced highest AUC (adjusted for age and sex) to distinguish multiple sclerosis from controls cases (sCD27: Test AUC 0.85; neurofilament light: Test AUC 0.85; Table 3 and Additional file 1: Table S4). Addition of further CSF biomarkers to the model incrementally improved the AUC up to a combination of five CSF biomarkers: Ch3L1 + Tumour necrosis factor receptor 1 (TNFR1) + sCD27 + C9 + TCC (Train/Test AUC 0.96/0.94; Table 3 and Additional file 1: Table S6).

**Table 3:** Optimal models to predict clinical multiple sclerosis versus non-multiple sclerosis status

Category	Biomarkers	*N*	Markers	AUC train	AUC test
CSF	TCC + C9 + Ch3L1 + TNFR1 + sCD27	157	5	0.96	0.94
Serum	iC3b + CCL27 + osteopontin + MCP1	148	4	0.93	0.91
CSF/serum	CSF[Ch3L1+ TNFR1 + sCD27] + serum[osteopontin + MCP1]	157	5	0.97	0.97

Osteopontin and neurofilament light were the single serum biomarkers with highest AUC to distinguish multiple sclerosis from control cases (osteopontin: Test AUC 0.83; neurofilament light: Test AUC 0.80). Addition of further serum biomarkers to the model incrementally improved AUC up to a combination of four serum biomarkers: osteopontin + iC3b + monocyte chemoattractant protein 1 (MCP1) + CCL27 (Train/Test AUC 0.93/0.91, Table 3 and Additional file 1: Table S6). When combining serum and CSF biomarkers in a single model, the AUC plateaued at a combination of five markers: CSF[chitinase-3-like-1 + TNFR1 + sCD27] + serum[osteopontin + MCP1] (Train/Test AUC 0.97/0.95; Table 3 and Additional file 1: Table S6 and S7).

**Fig. 2:**
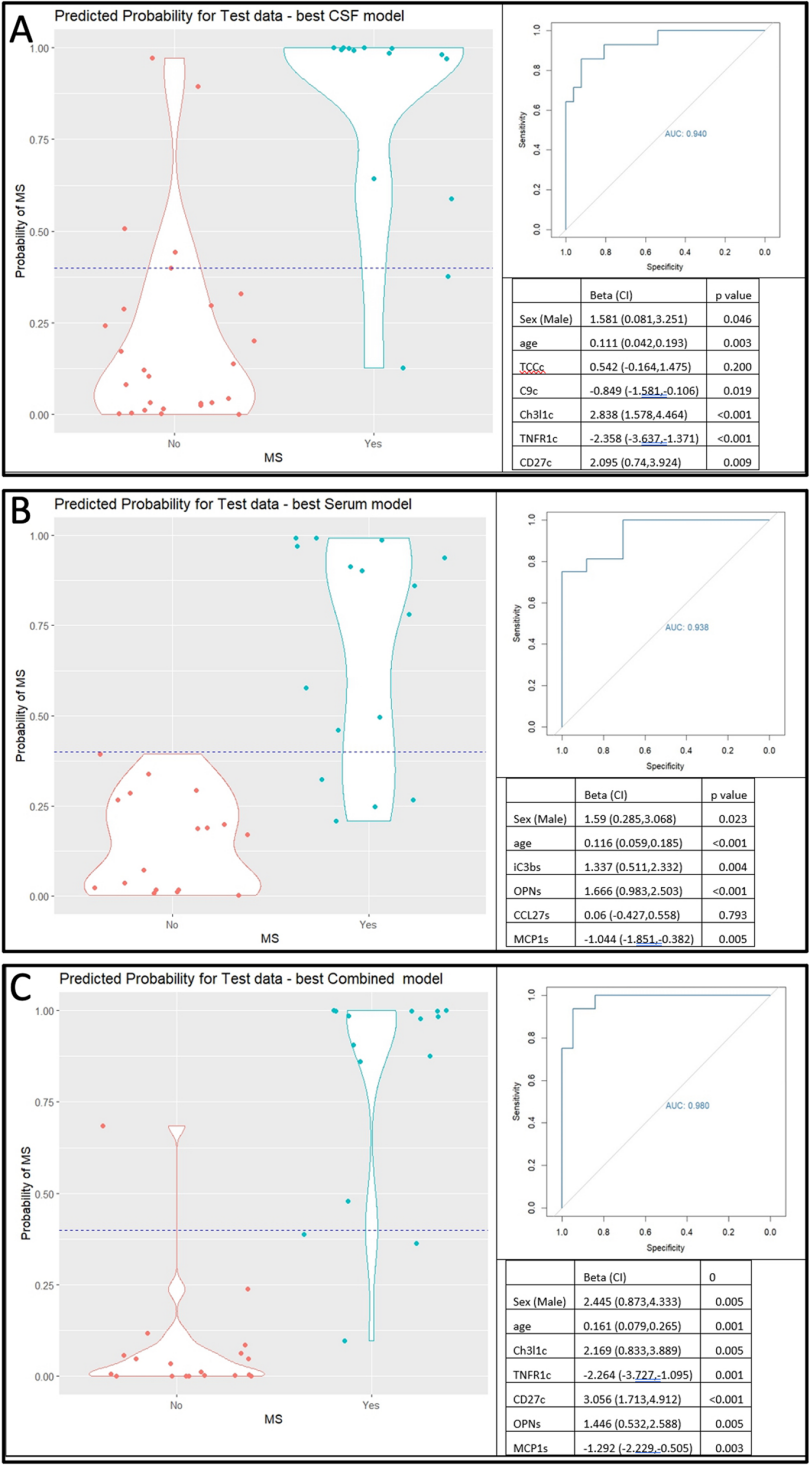
Logistic regression model output of multiple sclerosis versus non-multiple sclerosis. Illustration of the model expressed according to best combination of **A** CSF, **B** serum, and **C** combined CSF and serum biomarkers. Top left: violin plot illustrating the predicted probability of multiple sclerosis (*y*-axis) by cohort (*x*-axis), according to the optimum biomarker model adjusted for sex and age. The width of the violin plots indicates the relative number of probabilities at the probability. Horizontal dashed line illustrates an arbitrary cut-off value that could be applied to distinguish MS from non-MS cases, where red dots above the line indicate false positives and blue dots below the line false negatives. Top right: receiver operator curve illustrating performance of the optimum model. Bottom right: regression table indicating the magnitude and direction of effect of each variable. Note: a cross-validation approach was taken whereby the cohort was randomly divided into four roughly equally sized and distinct groups (see Methods), which were separately used as “training datasets” to produce a model using roughly 75% of the data, which was then tested on the remaining approximately 25% of the data (the “test dataset”). This was repeated 4 times to generate a mean test AUC to determine the optimum combination of biomarkers. The ROC curve, regression table and AUCs shown here were produced using one of the train/test sub-cohorts

Figure 2 illustrates example violin plots and ROC curves for the optimum combinations of CSF, serum and mixed CSF + serum for their ability to distinguish MS from non-MS cases. There were no obvious patterns of clinical or demographic characteristics of non-MS cases who were misclassified as controls (data not shown). Re-running modelling for the optimum combinations of biomarkers in CSF, serum and CSF and serum 1000 times in random selections of Train and Test data demonstrated mean AUCs > 0.9 for all models (Additional file 1: Fig. S1).

Given the difference in age and sex between control and MS cohorts, we performed a sensitivity analysis repeating the modelling of AUC using biomarker values that were corrected for the potential effects of age and sex (measured in the controls). This did not lead to any substantial difference in outcomes (Additional file 1: Table S8).

### Cox regression modelling predicts time to next relapse

We used univariate Cox regression, adjusted for age and sex and censored for commencement of disease modifying therapy (since disease-modifying therapy is expected to mask new relapses). Thirty-six (47%) people with multiple sclerosis experienced a relapse during follow-up and prior to disease modifying therapy commencement. The CSF biomarkers with highest concordance for predicting time to next relapse were vitamin D binding protein (concordance 0.65, *p* = 0.083) and neurofilament light (concordance 0.61, *P* = 0.42; Additional file 1: Table S9). The addition of further biomarkers in a multivariate model improved concordance up to a combination of six biomarkers: vitamin D binding protein + neurofilament light + CXCL12 + Factor B + sCD27 + MCP1 (concordance 0.72; *P* = 0.075; Table 4 and Additional file 1: Table S9).

**Table 4:** Optimal models to predict clinical outcomes

Outcome	Category	Biomarkers	Markers	*N*	Events	Concordance	*P* value
Time to next relapse	CSF	vitamin D binding protein + CXCL12 + Factor B + CD27 + MCP1 + neurofilament light	6	68	34	0.72	0.075
	Serum	C1inh/C1s + C3 + Factor H + CXCL12 + CD27 + vitamin D binding protein	6	67	31	0.72	0.02
	Serum/CSF	CSF[vitamin D binding protein + Factor I + C1inh/C1s] + serum[Factor B + IL4 + C1inh/C1s]	6	63	29	0.80	< 0.001
Time to EDSS 6	CSF	neurofilament light + TNFR1 + iC3b + CXCL12	4	68	17	0.93	< 0.001
	Serum	C1inh/C1s + CCL27 + Factor I + osteopontin	4	65	15	0.94	< 0.001
	Serum/CSF	CSF[C9 + neurofilament light] + serum[chitinase-3-like-1 + CCL27 + vitamin D binding protein + C1inh/C1s]	6	61	14	0.98	< 0.001

In univariate analysis adjusted for age and sex, and censored for commencement of disease modifying therapy, the serum biomarkers with highest concordance for predicting time to next relapse were C5 (concordance 0.61, *P* = 0.04) and C1inh/C1s (concordance 0.60, *P* = 0.05; Additional file 1: Table S9). The addition of further biomarkers to the model improved concordance up to a combination of six serum markers: C1inh/C1s + C3 + Factor H + CXCL12 + sCD27 + vitamin D binding protein (concordance 0.72, *P* = 0.02, Table 4 and Additional file 1: Table S10). Combining CSF and serum markers together in a single model showed concordance for time to next relapse prediction that improved up to a combination of six markers: CSF[vitamin D binding protein + Factor I + C1inh/C1s] + serum[Factor B + IL4 + C1inh/C1s] (concordance 0.80, *P* < 0.001).

### Cox regression modelling predicts time to disability

Four people with multiple sclerosis had already reached EDSS 6 by the time of sampling so were excluded from the analysis. Nineteen of the remaining 73 (26%) people with multiple sclerosis reached EDSS 6 during follow-up. In univariate Cox regression, adjusted for age and sex, the CSF biomarkers with highest concordance for predicting time to EDSS 6 were neurofilament light (concordance 0.92, *P* = 0.047) and C1inh/C1s (concordance 0.91, *P* = 0.79; Additional file 1: Table S11). The addition of biomarkers in a multivariate model improved concordance up to a combination of four biomarkers: neurofilament light + TNFR1 + iC3b + CXCL12 (concordance 0.93; *p* < 0.001; Table 4 and Additional file 1: Table S10).

**Fig. 3:**
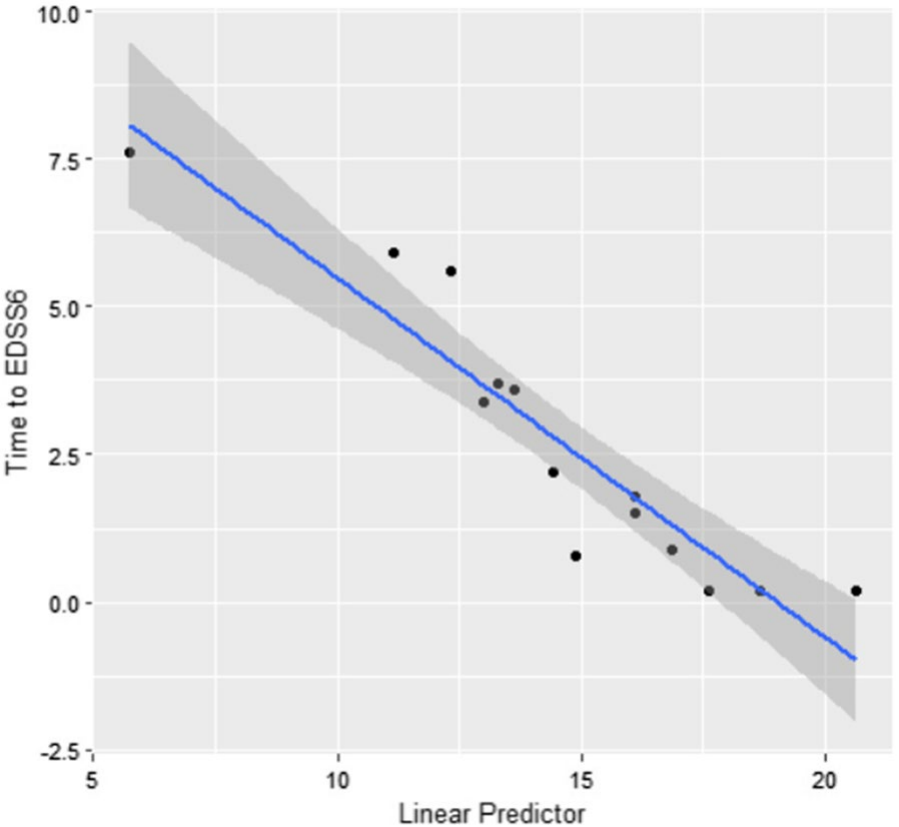
Plot of the linear risk predictor from the optimum combined biomarker model and time to EDSS 6. The optimum combined model contained age, sex and CSF[C9 + neurofilament light] + serum[chitinase-3-like-1 + CCL27 + vitamin D binding protein + C1inh/C1s]

In univariate analysis adjusted for age and sex, the serum biomarkers with highest signal for concordance for predicting time to EDSS 6 were C1inh/C1s (concordance 0.90, *P* = 0.91) and sCD27 (concordance 0.86, *P* = 0.86). The addition of further biomarkers to the model improved concordance up to a combination of four serum markers: CCL27 + Factor I + osteopontin + C1inh/C1s (concordance 0.94, *P* < 0.001; Table 4 and Additional file 1: Table S10). Combining CSF and serum markers together in a single model showed concordance for EDSS 6 prediction that improved up to a combination of six markers: CSF[C9 + neurofilament light] + serum[chitinase-3-like-1 + CCL27 + vitamin D binding protein + C1inh/C1s] (concordance 0.98; *P* < 0.001; Fig. 3, Additional file 1: Table S10).

## Discussion

Biomarkers hold promise to expedite diagnosis and stratify the risk of adverse multiple sclerosis prognosis, which would aid personalisation of therapy. Candidate multiple sclerosis biomarkers have been proposed but are often studied in isolation, and comparing studies that have used different clinical outcomes is challenging [12]. Given the complexity of multiple sclerosis biology, a combination of markers may improve predictions versus any marker in isolation. Here we have shown that combinations of up to six biomarkers measured in CSF or serum provide incremental improvements in the prediction of diagnosis and prognosis in multiple sclerosis (Fig. 2). Importantly, we found that predictions using combinations of blood-based biomarkers rivalled those using CSF markers, offering promise for less invasive approaches to monitoring and personalising multiple sclerosis care.

Neurofilament-light is a well-established candidate biomarker in multiple sclerosis and in our study was among the optimum single biomarkers to distinguish multiple sclerosis from control cases in both serum and CSF. Meta-analysis and systematic review have both highlighted evidence supporting neurofilament light as a marker to predict future multiple sclerosis disease activity, brain atrophy and to monitor treatment response to disease modifying therapies [17, 55]. Our model confirmed the predictive power of neurofilament light in CSF to distinguish multiple sclerosis from control cases (AUC=0.85). With the addition of further CSF markers, CSF NfL did not feature in our final predictive model (C9, chitinase-3-like-1, TNFR1, sCD27; AUC to 0.94) although this may have been affected by the relationship of NfL with age, since CSF NfL did feature in a model adjusted for the effects of age and sex within controls (Fig. 2, Table 3, Additional file 1: Table S8). The limitations of ELISA in detecting low concentrations of neurofilament light in blood have been overcome using highly sensitive SiMoA technology, and some clinics are starting to incorporate neurofilament light into practice [8]. In our study, while serum neurofilament light was one of the top biomarkers to distinguish multiple sclerosis from control cases, it did not feature in the final serum model of diagnosis. Some studies have demonstrated utility in combining neurofilament light with GFAP [18], which can also be measured using SiMoA. We used an ELISA assay for GFAP and were unable to adequately detect GFAP in CSF; serum GFAP detected using ELISA did not feature in any of our predictive models.

Chitinase-3-like-1 featured in CSF predictive models of multiple sclerosis versus control status in our cohort, and also as a serum marker in the combined CSF/serum model predicting time to EDSS 6. Chitinase-3-like-1 is a marker of macrophage and astrocyte activation and is potentially neurotoxic [19]. It has been found to be elevated in other studies of multiple sclerosis CSF [20–22], and has shown promise in predicting conversion from CIS to multiple sclerosis and future multiple sclerosis disease activity [22, 23]. Other markers that predicted multiple sclerosis versus control status in our study were chemokines/cytokines known to promote a pro-inflammatory milieu such as osteopontin, MCP1, CCL27, TNFR1 and soluble CD27. Osteopontin is a cytokine produced by a range of cells including macrophages, lymphocytes and dendritic cells, providing cross talk between the innate and adaptive immune system. Osteopontin regulates the differentiation of pro-inflammatory lymphocytes, inhibits apoptosis of inflammatory cells [24], and has a role in microglia-mediated synaptic engulfment [25]. CCL27 and MCP1 (CCL2) are both chemokines involved in regulating migration of macrophages/monocytes [26]. MCP1 is abundantly expressed by microglia located at the active rim of multiple sclerosis lesions [27], and is thought to play a role in multiple sclerosis pathogenesis [28], although we and others found it to be significantly lower in serum and CSF of people with multiple sclerosis than controls [29]. CCL27 induces the homing of memory T cells to sites of inflammation and has been found by others to be elevated in the serum of people with multiple sclerosis [30]. Tumour necrosis factor alpha (TNFα) is recognised as a key function in autoimmune disease, where excessive activation of TNFα mediates cytotoxic and pro-inflammatory responses via TNFR1 [31]. CD27 is a T-cell activation marker, whose soluble form has been shown by others to be significantly elevated in people with multiple sclerosis [32], and predictive of future multiple sclerosis disease activity [33], including transition from CIS to multiple sclerosis [34].

CSF biomarkers that combined to predict time to next relapse also included neurofilament light and MCP1. Vitamin D binding protein, a regulator of the distribution, stability and bioavailability of vitamin D, was one of the markers whose expression differed significantly between multiple sclerosis and control cases in serum and also featured in the CSF and serum models predicting relapse. There is evidence for an immunomodulatory role of vitamin D in multiple sclerosis [35]. Other studies have demonstrated some utility of vitamin D binding protein in distinguishing people with multiple sclerosis versus controls [36, 37], or risk of developing multiple sclerosis [38]. Vitamin D binding protein has been shown to be expressed on spinal cord neurons, pia mater and grey matter within the brains of people with multiple sclerosis, and in an animal model of multiple sclerosis, high vitamin D binding protein appeared to mitigate beneficial effects of vitamin D3 supplementation and inhibit recovery [37]. CXCL12, a chemoattractant protein for T cells as well as monocytes appeared in the CSF and serum models predicting relapse, and the CSF model predicting time to EDSS 6. CXCL12 has been found highly expressed in active multiple sclerosis lesions and appears to play a role in enhancing the inflammatory response in multiple sclerosis [39].

Our models also demonstrated evidence of complement activation and consumption. In our cohort, significantly differences in the concentration of several complement proteins between the CSF and serum of multiple sclerosis versus controls suggested dysregulation of this pathway in multiple sclerosis. The complement molecules TCC (terminal complement protein) and iC3b (activation product) featured in our final model to predict multiple sclerosis status. C1inh/C1s complex also featured prominently in the prediction of relapses and disability in our cohort. This is an indication of activation of the classical pathway of the complement cascade [40], and in line with our earlier work suggesting this is relevant to multiple sclerosis biology [16]. C3 and Factor H both featured in our serum model predicting relapses. We have previously shown both to be present at high levels in multiple sclerosis lesions [41], while others have shown these complement proteins to be elevated in the blood and CSF of people with multiple sclerosis versus controls, and the be relevant in predictions of disability outcomes [42, 43]. Overall, our data support previous findings that imply ongoing local and systemic complement dysregulation in multiple sclerosis [16, 44].

Others have explored the potential utility of combining more than one protein biomarker to predict multiple sclerosis diagnosis or prognosis. Lucchini et al. measured a panel of 8 protein candidates in the CSF of people with multiple sclerosis or controls. They found the combination of chitinase-3-like-1, CXCL10, CXCL12, CXCL13 increased the AUC for predicting conversion to clinically definite multiple sclerosis after first attack above any single biomarker in isolation [22]. Bielekova et al*.* [45] also found that the combination of CSF IL-12/IL-23p40, CXCL13 and IL-8 in CSF was more predictive of multiple sclerosis versus control status than any of these markers in isolation.

There are some practical considerations around combining biomarkers in clinical practice. Some biomarkers were not statistically significant in univariate analyses, but still provided a small differential addition to the final model. Conversely, some of the biomarkers that were most discriminatory in univariate analysis autocorrelated to some extent and therefore did not all appear in final combination models. There may be a balance between selecting the model that was statistically optimal versus a choosing smaller combination of markers that gives similar predictive value but is simpler to measure in multiplex (Additional file 1: Table S7 and S9). While optimum models sometimes combined CSF and serum markers, there are practical advantages in using a single sample type, ideally serum, which can be serially sampled more easily than CSF.

The present study is subject to some limitations or caveats. Using research operating procedures, we aimed for all blood and CSF samples to reach the freezer within 2 h. Variations in sample handling in routine clinical practice may introduce the risk of degradation of small molecules such as complement [46]. The demographics of our multiple sclerosis and control group differed, which could affect the results we are observing, even though we adjusted for sex and age in all analyses. Body mass index (BMI) data were unavailable for this cohort, so were not included, despite emerging evidence that correction of some biomarkers for BMI improves correlations [47]. Our cohort was too small to explore biomarker signatures of different multiple sclerosis subtypes. We included all people with multiple sclerosis in the analysis of time to relapse, even though people with progressive disease are somewhat less likely to experience relapses. While over-fitting of statistical models is a potential limitation, our test and train AUCs were very similar which suggests this was not the case in our study. However, the main caveat to our results is that this represents “discovery” work, which means that conclusions on the potential utility of these combinations could only be recognised with validation studies in independent cohorts.

In conclusion, using well-optimised assays for 24 candidate protein biomarkers in the blood and 20 CSF we have demonstrated that combination models showed better prediction of multiple sclerosis diagnosis and prognosis than single biomarkers. We also demonstrated for the first time that combination serum models rivalled those of CSF, holding promise for a non-invasive approach. This study supports the premise that combining several biomarkers in a single test will aid diagnostic and prognostic accuracy in the future. The utility of our biomarker models can only be established by robust validation in different and varied cohorts.

### Supplementary Information

**Additional file 1: Table S1.** An overview of the assays used for this study. BDNF: brain-derived neurotrophic factor; CCL27: CC motif chemokine ligand 27; CRP: C-reactive protein; CXCL: C-X-C motif chemokine ligand; GFAP: glial fibrillary acidic protein; IL: interleukin; LIF: leukaemia inhibitory factor; MCP1: monocyte chemoattractant protein 1; TGF-β: transforming growth factor beta; TNFα: tumour necrosis factor alpha; TNFR1: tumour necrosis factor receptor 1; TCC: terminal complement complex, VDBP: vitamin D binding protein. **Table S2.** Rates of missing or imputed data in assay results. **Table S3.** Characteristics of 4 test/ train cohorts. **Table S4.** Comparison of CSF biomarkers between multiple sclerosis and non-multiple sclerosis groups. Mann–Whitney comparisons, corrected for multiple comparisons using Benjamini–Hochberg procedure. **Table S5.** Comparison of serum biomarkers between multiple sclerosis and non-multiple sclerosis groups Mann–Whitney comparisons corrected for multiple comparisons using Benjamini–Hochberg procedure. **Table S6.** A progression from single through combinations of multiple biomarkers to predict multiple sclerosis versus non-multiple sclerosis status. **Table S7.** Breakdown of the Train / Test results for the combined CSF & serum modelling of MS versus non-MS status. Mean AUC values were ordered from lowest to highest, and the optimum model was selected when addition of a further analyte resulted in an AUC increase < 0.01. **Table S8.** Sensitivity analysis: all biomarker concentrations were corrected for age and sex according to a linear model generated in control samples. A progression from single through combinations of multiple biomarkers to predict multiple sclerosis versus non-multiple sclerosis status. **Table S9.** Concordance of biomarkers in predicting time to next relapse in univariate analysis (adjusted for sex and age). **Table S10.** A progression from single through combinations of multiple biomarkers to predict time to relapse and time to disability, adjusted for age and sex. **Table S11.** Concordance of biomarkers in predicting time to EDSS 6 in univariate analysis (adjusted for sex, age and disease modifying therapy). **Fig. S1.** The plots show the 3 models below, run on 1000 random selection of (approx 75%) Train and (approx. 25%) Test data. The left hand plot is the range of AUC’s from the Train data when used also as Test and the right-hand plot shows the range of AUC’s when Test data are used as test.

## Data Availability

The data that support the findings of this study, including code used in analyses, are available in the following repository: https://github.com/EmmaTallantyre/Biomarkers_MS_2024.
