# Supplementary material for: Combination protein biomarkers predict multiple sclerosis diagnosis and outcomes
Source: J Neuroinflammation. 2024 Feb 17;21:52. doi: 10.1186/s12974-024-03036-4 (PMC10874571; doi:10.1186/s12974-024-03036-4)
Supplement: Supplementary file 1 — Additional file 1: Table S1. An overview of the assays used for this study. BDNF: brain-derived neurotrophic factor; CCL27: CC motif chemokine ligand 27; CRP: C-reactive protein; CXCL: C-X-C motif chemokine ligand; GFAP: glial fibrillary acidic protein; IL: interleukin; LIF: leukaemia inhibitory factor; MCP1: monocyte chemoattractant protein 1; TGF-β: transforming growth factor beta; TNFα: tumour necrosis factor alpha; TNFR1: tumour necrosis factor receptor 1; TCC: terminal complement complex, VDBP: vitamin D binding protein. Table S2. Rates of missing or imputed data in assay results. Table S3. Characteristics of 4 test/ train cohorts. Table S4. Comparison of CSF biomarkers between multiple sclerosis and non-multiple sclerosis groups. Mann–Whitney comparisons, corrected for multiple comparisons using Benjamini–Hochberg procedure. Table S5. Comparison of serum biomarkers between multiple sclerosis and non-multiple sclerosis groups Mann–Whitney comparisons corrected for multiple comparisons using Benjamini–Hochberg procedure. Table S6. A progression from single through combinations of multiple biomarkers to predict multiple sclerosis versus non-multiple sclerosis status. Table S7. Breakdown of the Train / Test results for the combined CSF & serum modelling of MS versus non-MS status. Mean AUC values were ordered from lowest to highest, and the optimum model was selected when addition of a further analyte resulted in an AUC increase < 0.01. Table S8. Sensitivity analysis: all biomarker concentrations were corrected for age and sex according to a linear model generated in control samples. A progression from single through combinations of multiple biomarkers to predict multiple sclerosis versus non-multiple sclerosis status. Table S9. Concordance of biomarkers in predicting time to next relapse in univariate analysis (adjusted for sex and age). Table S10. A progression from single through combinations of multiple biomarkers to predict time to relapse and time to disabil [file 12974_2024_3036_MOESM1_ESM.docx]

**Supplementary Material**

| **Marker** | **Manufacturer** | **Catalogue number** | **Dilution serum** | **Dilution CSF** |
| --- | --- | --- | --- | --- |
| neurofilament light | Uman diagnostics | 10-7001 | n/a (SiMoA) | 1 in 2 |
| IL-12/IL-23p40** | R&D Systems, UK | DY1240 | Neat | Neat |
| Osteopontin | R&D Systems, UK | DY1433 | Neat | 1 in 100 |
| CRP | R&D Systems, UK | DY1707 | 1 in 16000 | 1 in 16 |
| IL-4 | R&D Systems, UK | DY204 | Neat | Neat |
| IL-6** | R&D Systems, UK | DY206 | Neat | Neat |
| IL-8 | R&D Systems, UK | DY208 | Neat | Neat |
| TNFα | R&D Systems, UK | DY210 | Neat | Neat |
| IL-10 | R&D Systems, UK | DY217B | Neat | Neat* |
| TNFR1 (s) | R&D Systems, UK | DY225 | Neat | Neat |
| TGF-β** | R&D Systems, UK | DY240 | Neat | Neat |
| BDNF | R&D Systems, UK | DY248 | 1 in 100 | Neat* |
| GFAP | R&D Systems, UK | DY2594 | Neat | Neat* |
| CHi3L1 | R&D Systems, UK | DY2599 | 1 in 50 | 1 in 160 |
| MCP-1 | R&D Systems, UK | DY279 | Neat | Neat |
| IFNγ** | R&D Systems, UK | DY285 | Neat | Neat |
| IL-18 | R&D Systems, UK | DY318 | Neat | Neat |
| CXCL12 | R&D Systems, UK | DY350 | 1 in 10 | Neat |
| CCL27 | R&D Systems, UK | DY376 | Neat | Neat* |
| VDBP | R&D Systems, UK | DY3778 | 1 in 100000 | 1 in 200 |
| Soluble CD27 | R&D Systems, UK | DY382 | 1 in 5 | Neat |
| LIF | R&D Systems, UK | DY7734 | Neat | Neat* |
| CXCL13 | R&D Systems, UK | DY801 | Neat | Neat* |
| TCC | in-house | n/a | 1 in 50 | Neat |
| Factor H | in-house | n/a | 1 in 4000 | 1 in 40 |
| iC3b | in-house | n/a | 1 in 100 | 1 in 2 |
| Factor B | in-house | n/a | 1 in 500 | 1 in 4 |
| C5 | in-house | n/a | 1 in 500 | 1 in 4 |
| C9 | in-house | n/a | 1 in 2000 | 1 in 8 |
| C3 | in-house | n/a | 1 in 20000 | 1 in 320 |
| Factor I | in-house | n/a | 1 in 250 | Neat |
| C1inhibitor/C1s | Hycult Biotech, UK | HK399 | 1 in 100 | 1 in 2 |

**Supplementary Table 1**: **An overview of the assays used for this study.** BDNF: brain derived neurotrophic factor; CCL27: CC motif chemokine ligand 27; CRP: C reactive protein; CXCL: C-X-C motif chemokine ligand; GFAP: glial fibrillary acidic protein; IL: interleukin; LIF: leukaemia inhibitory factor; MCP1: monocyte chemoattractant protein 1; TGF-β: transforming growth factor beta; TNFα: tumour necrosis factor alpha; TNFR1: tumour necrosis factor receptor 1; TCC: terminal complement complex, VDBP: vitamin D binding protein.

*=not detected in CSF, **=not detected in most samples, serum or CSF.

| **Marker** | **Missing data CSF* (n)** | | **Missing data serum* (n)** | | **Imputed data CSF ^+^(n)** | | **Imputed data serum^+^ (n)** | | **Lowest**  **value** | **Half lowest**  **value** |
| --- | --- | --- | --- | --- | --- | --- | --- | --- | --- | --- |
|  | **MS** | **Non-NS** | **MS** | **Non-NS** | **MS** | **Non-NS** | **MS** | **Non-NS** |  |  |
| TCC | 0 | 0 | 0 | 0 | 12 | 27 | 0 | 0 | 0.0038 ug/ml | 0.0019 ug/ml |
| iC3b | 0 | 0 | 0 | 0 | 23 | 26 | 0 | 0 | 0.0038 ug/ml | 0.0019 ug/ml |
| C5 | 0 | 0 | 0 | 0 | 4 | 31 | 1 | 0 | 0.0098 ug/ml | 0.0049 ug/ml |
| sCD27 | 0 | 0 | 0 | 0 | 19 | 32 | 4 | 8 | 0.0020 ng/ml | 0.0010 ng/ml |
| IL-4 | 0 | 0 | 0 | 0 | 1 | 6 | 0 | 1 | 1.04 pg/ml | 0.52 pg/ml |
| CXCL13 | - | - | 0 | 0 | - | - | 3 | 2 | 1.21 pg/ml | 0.61 pg/ml |
| CXCL12 | 0 | 0 | 0 | 0 | 0 | 2 | 0 | 0 | 0.021 ng/ml | 0.011 ng/ml |
| C9 | 0 | 0 | 0 | 0 | 2 | 0 | 0 | 2 | 0.034 ug/ml | 0.017 ug/ml |
| NfL | 5 | 0 | 3 | 3 | 0 | 0 | 0 | 0 | N/a | N/a |
| CCL27 | - | - | 0 | 5 | - | - | 2 | 1 | 0.0083ng/ml | 0.0042ng/ml |
| C1Inh/C1s | 5 | 0 | 8 | 2 | 0 | 0 | 0 | 0 | N/a | N/a |
| IL8 | 0 | 0 | - | - | 3 | 7 | - | - | 1.47pg/ml | 0.74pg/ml |

**Supplementary Table 2** **Rates of missing or imputed data in assay results.** C1inh/C1s: C1-inibitor/C1s complex; CCL27: CC motif chemokine ligand 27; CXCL: C-X-C motif chemokine ligand; GFAP: IL: interleukin; LIF: leukaemia inhibitory factor; N/a: not applicable; NfL: neurofilament light; sCD27: soluble CD27; TCC: terminal complement complex. *missing data arose where sample was consumed and therefore unavailable for analysis. ^+^ imputed data was used where sample was analysed but no analyte was detectable. Analytes not listed in the table had neither missing, nor imputed data. – denotes that analyte was not measured in sample type

**Supplementary Material**

| **Cohort** |  | **MS (n (%))** | **Non-MS (n (%))** | **Female (n (%))** | **Male (n (%))** | **RRMS/Tot ((n (%))** | **PMS/Tot ((n (%))** | **Age (mean (sd))** | **Total** |
| --- | --- | --- | --- | --- | --- | --- | --- | --- | --- |
|  | **1** | 19 (54.3%) | 16 (45.7%) | 23 (65.7%) | 12 (34.3%) | 10/19 (52.6%) | 9/19 (47.4%) | 38.7 (11.6) | **35** |
|  | **2** | 16 (45.7%) | 19 (54.3%) | 27 (77.1%) | 8 (22.9%) | 14/16 (87.5%) | 2/16 (12.5%) | 37.7 (11.9) | **35** |
|  | **3** | 14 (35%) | 26 (65%) | 32 (80%) | 8 (20%) | 11/14 (78.6%) | 3/14 (21.4%) | 35 (13.4) | **40** |
|  | **4** | 28 (59.6%) | 19 (40.4%) | 36 (76.6%) | 11 (23.4%) | 22/28 (78.6%) | 6/28 (21.4%) | 38.4 (11.7) | **47** |
|  | **Total** | **77 (49%)** | **80 (51%)** | **118 (75.2%)** | **39 (24.8%)** | **57/77 (74.0%)** | **20/77 (26.0%)** | **37.5 (12.1)** | **157** |

**Supplementary Table 3:** **Characteristics of 4 test/ train cohorts.** Age, sex and Disease characteristics of the 4 cohorts used to test and train the biomarker models.

|  | **Markers** | ***P* Value** | **Adjusted *P* Value** | **Fold Change** | **Test Mean AUC from Non Adjusted Model** | **Test Mean AUC from Adjusted Model** |
| --- | --- | --- | --- | --- | --- | --- |
| 1 | sCD27 | 1.77E-06 | 1.94E-05* | 4.1347 | 0.7040 | 0.8514 |
| 2 | neurofilament light | 1.00E-05 | 8.81E-05* | 3.4369 | 0.8500 | 0.8487 |
| 3 | Chitinase-3-like-1 | 5.96E-10 | 2.62E-08* | 1.6291 | 0.8019 | 0.8336 |
| 4 | osteopontin | 3.47E-05 | 0.0003* | 1.3342 | 0.6698 | 0.7881 |
| 5 | C5 | 0.0003 | 0.0018* | 1.6996 | 0.6934 | 0.8012 |
| 6 | iC3b | 0.0040 | 0.0157* | 2.6172 | 0.6011 | 0.7998 |
| 7 | C9 | 0.0043 | 0.0157* | 0.7632 | 0.6860 | 0.7744 |
| 8 | TCC | 0.0063 | 0.0214* | 1.4723 | 0.6302 | 0.7685 |
| 9 | C3 | 0.0299 | 0.0800 | 1.1828 | 0.6041 | 0.7690 |
| 10 | vitamin D binding protein | 0.0357 | 0.0874 | 1.3282 | 0.6392 | 0.7817 |
| 11 | IL4 | 0.0401 | 0.0929 | 1.3387 | 0.6537 | 0.7889 |
| 12 | CRP | 0.0783 | 0.1640 | 0.5980 | 0.6060 | 0.7626 |
| 13 | IL8 | 0.0855 | 0.1710 | 1.2839 | 0.5911 | 0.7667 |
| 14 | Factor I | 0.2715 | 0.4182 | 1.0765 | 0.5160 | 0.7693 |
| 15 | MCP1 | 0.3221 | 0.4429 | 0.8455 | 0.5292 | 0.7878 |
| 16 | TNFR1 | 0.4515 | 0.5843 | 0.9543 | 0.4916 | 0.7698 |
| 17 | C1-inhitor | 0.7666 | 0.9117 | 1.0449 | 0.6233 | 0.7611 |
| 18 | Factor H | 0.8196 | 0.9285 | 1.0147 | 0.5731 | 0.7603 |
| 19 | Factor B | 0.8652 | 0.9285 | 0.9845 | 0.5357 | 0.7588 |
| 20 | CXCL12 | 0.9005 | 0.9396 | 0.9724 | 0.5710 | 0.7709 |

**Supplementary Table 4 Comparison of CSF biomarkers between multiple sclerosis and non-multiple sclerosis groups**. Mann-Whitney comparisons, corrected for multiple comparisons using **Benjamini-Hochberg** procedure. CRP: C reactive protein; CXCL: C-X-C motif chemokine ligand; IL: interleukin; MCP1: monocyte chemoattractant protein 1; TNFR1: tumour necrosis factor receptor 1; TCC: terminal complement complex. * statistically significant at p<0.05.

|  | **Markers** | **p Value** | **Adjusted p Value** | **Fold Change** | **Test Mean AUC from Non Adjusted Model** | **Test Mean AUC from Adjusted Model** |
| --- | --- | --- | --- | --- | --- | --- |
| 1 | osteopontin | 1.10E-07 | 2.42E-06* | 1.5425 | 0.7225 | 0.8336 |
| 2 | Factor B | 1.08E-06 | 1.59E-05* | 0.7801 | 0.7459 | 0.7970 |
| 3 | vitamin D binding protein | 0.0017 | 0.0088* | 0.8176 | 0.6499 | 0.7874 |
| 4 | C5 | 0.0018 | 0.0088* | 1.3139 | 0.6459 | 0.7737 |
| 5 | iC3b | 0.0040 | 0.0157* | 1.3298 | 0.6161 | 0.7700 |
| 6 | CRP | 0.0074 | 0.0224* | 0.4549 | 0.6717 | 0.7735 |
| 7 | neurofilament light | 0.0076 | 0.0224* | 1.5943 | 0.6926 | 0.8057 |
| 8 | MCP1 | 0.0309 | 0.0800 | 0.8385 | 0.5616 | 0.7791 |
| 9 | BDNF | 0.0482 | 0.1061 | 1.2202 | 0.6028 | 0.7714 |
| 10 | IL18 | 0.1024 | 0.1959 | 0.8816 | 0.6362 | 0.7557 |
| 11 | Chi3L1 | 0.1144 | 0.2097 | 1.2097 | 0.6078 | 0.7563 |
| 12 | C3 | 0.1776 | 0.3126 | 0.9479 | 0.6282 | 0.7677 |
| 13 | TNFR1 | 0.1973 | 0.3339 | 0.8733 | 0.5794 | 0.7672 |
| 14 | Factor I | 0.2208 | 0.3599 | 0.9373 | 0.6085 | 0.7737 |
| 15 | CXCL13 | 0.2756 | 0.4182 | 3.0663 | 0.5222 | 0.7731 |
| 16 | C9 | 0.2950 | 0.4327 | 0.8991 | 0.5845 | 0.7677 |
| 17 | IL4 | 0.3108 | 0.4412 | 6.9503 | 0.5547 | 0.7637 |
| 18 | CXCL12 | 0.5245 | 0.6594 | 1.5364 | 0.5136 | 0.7718 |
| 19 | TCC | 0.7296 | 0.8917 | 1.0289 | 0.5503 | 0.7572 |
| 20 | Factor H | 0.8357 | 0.9285 | 0.9913 | 0.5427 | 0.7635 |
| 21 | CCL27 | 0.8643 | 0.9285 | 1.0547 | 0.5879 | 0.7686 |
| 22 | C1inh/C1s | 0.9182 | 0.9396 | 1.0051 | 0.5754 | 0.7869 |
| 23 | sCD27 | 0.9627 | 0.9627 | 1.0518 | 0.5286 | 0.7538 |

**Supplementary Table 5 Comparison of serum biomarkers between multiple sclerosis and non-multiple sclerosis groups** Mann-Whitney comparisons corrected for multiple comparisons using **Benjamini-Hochberg** procedure. BDNF: brain derived neurotrophic factor; CCL27: CC motif chemokine ligand 27; Chi3L1: chitinase-3-like-1; CRP: C reactive protein; CXCL: C-X-C motif chemokine ligand; GFAP: glial fibrillary acidic protein; IL: interleukin; LIF: leukaemia inhibitory factor; MCP1: monocyte chemoattractant protein 1; TGF-β: transforming growth factor beta; TNFα: tumour necrosis factor alpha; TNFR1: tumour necrosis factor receptor 1; TCC: terminal complement complex, * statistically significant at p<0.05.

| **Category** | **Biomarkers** | **N** | **Markers** | **AUC Train** | **AUC Test** |
| --- | --- | --- | --- | --- | --- |
| CSF | Ch3l1 | 157 | 1 | 0.84 | 0.84 |
|  | Ch3l1 + TNFR1 | 157 | 2 | 0.91 | 0.90 |
|  | Ch3l1 + TNFR1 + sCD27 | 157 | 3 | 0.95 | 0.93 |
|  | TCC + Ch3L1 + TNFR1 + sCD27 | 157 | 4 | 0.95 | 0.94 |
|  | TCC + C9 + Ch3L1 + TNFR1 + sCD27 | 157 | 5 | 0.96 | 0.94 |
| Serum | OPN | 148 | 1 | 0.86 | 0.86 |
|  | iC3b + OPN | 148 | 2 | 0.90 | 0.89 |
|  | iC3b + OPN + MCP1 | 148 | 3 | 0.92 | 0.92 |
|  | iC3b + OPN + CCL27 + MCP1 | 148 | 4 | 0.93 | 0.91 |
| CSF/ serum combined | Ch3l1 + TNFR1 | 157 | 2 | 0.91 | 0.90 |
|  | Ch3L1 + TNFR1 + sCD27 | 157 | 3 | 0.95 | 0.93 |
|  | Ch3L1 + TNFR1 + sCD27 + OPN | 157 | 4 | 0.96 | 0.96 |
|  | Ch3l1 + TNFR1 + sCD27 + OPN + MCP1 | 157 | 5 | 0.97 | 0.97 |

**Supplementary Table 6 A progression from single through combinations of multiple biomarkers to predict multiple sclerosis versus non-multiple sclerosis status.** Ch3L1: chitinase-3-like-1; MCP1: monocyte chemoattractant protein 1; TCC: terminal complement complex, TNFR1: tumour necrosis factor receptor 1.

| Biomarkers | n | Markers | Train AUC1 | Test AUC1 | Train AUC2 | Test AUC2 | Train AUC3 | Test AUC3 | Train AUC4 | Test AUC4 | Mean  Train AUC | **Mean**  **Test AUC** |
| --- | --- | --- | --- | --- | --- | --- | --- | --- | --- | --- | --- | --- |
| Ch3l1c + TNFR1c + CD27c + OPNs + MCP1s | 157 | 5 | 0.9698 | 0.9638 | 0.9667 | 0.9803 | 0.9797 | 0.9670 | 0.9706 | 0.9699 | 0.9717 | **0.9703** |
| Ch3l1c + TNFR1c + CD27c + OPNs + TNFR1s | 157 | 5 | 0.9655 | 0.9507 | 0.9678 | 0.9770 | 0.9809 | 0.9560 | 0.9686 | 0.9737 | 0.9707 | **0.9643** |
| Ch3l1c + TNFR1c + CD27c + C9s + OPNs | 157 | 5 | 0.9693 | 0.9507 | 0.9567 | 0.9770 | 0.9721 | 0.9505 | 0.9588 | 0.9756 | 0.9642 | **0.9634** |
| Ch3l1c + TNFR1c + CD27c + OPNs + NfL | 151 | 5 | 0.9756 | 0.9243 | 0.9649 | 0.9688 | 0.9681 | 0.9800 | 0.9632 | 0.9733 | 0.9679 | **0.9616** |
| Ch3l1c + TNFR1c + CD27c + iC3bs + OPNs | 157 | 5 | 0.9803 | 0.9243 | 0.9661 | 0.9770 | 0.9709 | 0.9643 | 0.9696 | 0.9680 | 0.9717 | **0.9584** |
|  |  |  |  |  |  |  |  |  |  |  |  |  |
| Ch3l1c + TNFR1c + CD27c + OPNs | 157 | 4 | 0.9671 | 0.9375 | 0.9562 | 0.9704 | 0.9674 | 0.9505 | 0.9555 | 0.9643 | 0.9616 | **0.9557** |
| Ch3l1c + TNFR1c + CD27c + MCP1s | 157 | 4 | 0.9564 | 0.9408 | 0.9557 | 0.9605 | 0.9647 | 0.9368 | 0.9629 | 0.9586 | 0.9599 | **0.9492** |
| Ch3l1c + TNFR1c + CD27c + TNFR1s | 157 | 4 | 0.9483 | 0.9211 | 0.9479 | 0.9441 | 0.9583 | 0.9368 | 0.9498 | 0.9624 | 0.9511 | **0.9411** |
| Ch3l1c + TNFR1c + CD27c + iC3bs | 157 | 4 | 0.9574 | 0.9276 | 0.9468 | 0.9375 | 0.9536 | 0.9423 | 0.9535 | 0.9549 | 0.9528 | **0.9406** |
| TCCc + Ch3l1c + TNFR1c + CD27c | 157 | 4 | 0.9520 | 0.9243 | 0.9409 | 0.9507 | 0.9515 | 0.9203 | 0.9455 | 0.9568 | 0.9475 | **0.9380** |
|  |  |  |  |  |  |  |  |  |  |  |  |  |
| Ch3l1c + TNFR1c + CD27c | 157 | 3 | 0.9480 | 0.9178 | 0.9387 | 0.9507 | 0.9521 | 0.9121 | 0.9441 | 0.9586 | 0.9457 | **0.9348** |
| Ch3l1c + OPNc + TNFR1c | 157 | 3 | 0.9049 | 0.9441 | 0.9051 | 0.9572 | 0.9224 | 0.8846 | 0.9351 | 0.8797 | 0.9169 | **0.9164** |
| Ch3l1c + TNFR1c + OPNs | 157 | 3 | 0.9100 | 0.9605 | 0.9178 | 0.9375 | 0.9330 | 0.8571 | 0.9237 | 0.9060 | 0.9211 | **0.9153** |
| Ch3l1c + TNFR1c + MCP1s | 157 | 3 | 0.9098 | 0.9408 | 0.9102 | 0.9572 | 0.9312 | 0.8599 | 0.9294 | 0.8929 | 0.9202 | **0.9127** |
| Ch3l1c + CD27c + OPNs | 157 | 3 | 0.9423 | 0.8487 | 0.9256 | 0.9211 | 0.9300 | 0.9066 | 0.9016 | 0.9662 | 0.9249 | **0.9106** |
|  |  |  |  |  |  |  |  |  |  |  |  |  |
| Ch3l1c + TNFR1c | 157 | 2 | 0.8968 | 0.9342 | 0.8990 | 0.9276 | 0.9186 | 0.8379 | 0.9197 | 0.8816 | 0.9085 | **0.8953** |
| Ch3l1c + OPNs | 157 | 2 | 0.8623 | 0.8947 | 0.8745 | 0.8684 | 0.8907 | 0.7885 | 0.8655 | 0.8797 | 0.8732 | **0.8578** |
| NfLc + OPNs | 152 | 2 | 0.9025 | 0.8264 | 0.8640 | 0.8912 | 0.8812 | 0.8077 | 0.8640 | 0.8863 | 0.8779 | **0.8529** |
| Ch3l1c + MCP1s | 157 | 2 | 0.8394 | 0.9342 | 0.8546 | 0.8421 | 0.8895 | 0.7637 | 0.8448 | 0.8684 | 0.8571 | **0.8521** |
| Ch3l1c + iC3bs | 157 | 2 | 0.8400 | 0.9112 | 0.8530 | 0.8520 | 0.8760 | 0.7720 | 0.8481 | 0.8628 | 0.8543 | **0.8495** |
|  |  |  |  |  |  |  |  |  |  |  |  |  |
| Ch3l1c | 157 | 1 | 0.8198 | 0.9079 | 0.8334 | 0.8553 | 0.8651 | 0.7225 | 0.8267 | 0.8571 | 0.8362 | **0.8357** |
| CD27c | 157 | 1 | 0.8790 | 0.7697 | 0.8589 | 0.8651 | 0.8757 | 0.7473 | 0.8240 | 0.9455 | 0.8594 | **0.8319** |
| OPNs | 157 | 1 | 0.8354 | 0.8750 | 0.8414 | 0.8651 | 0.8657 | 0.7500 | 0.8468 | 0.8308 | 0.8473 | **0.8302** |
| NfLc | 152 | 1 | 0.8906 | 0.8160 | 0.8378 | 0.8807 | 0.8525 | 0.7088 | 0.8333 | 0.9116 | 0.8535 | **0.8293** |
| iC3bc | 157 | 1 | 0.7829 | 0.8421 | 0.7992 | 0.8076 | 0.8288 | 0.6841 | 0.7926 | 0.8271 | 0.8009 | **0.7902** |

**Supplementary Table 7** **Breakdown of the Train / Test results for the combined CSF & serum modelling of MS versus non-MS status**. Mean AUC values were ordered from lowest to highest, and the optimum model was selected when addition of a further analyte resulted in an AUC increase < 0.01. Top 5 models for each combination of ‘n’ analytes (biomarkers). In column 1, the suffix “c” denotes CSF biomarker and “s” denotes serum biomarker.

| **Category** | **Biomarkers** | **N** | **Markers** | **AUC Train** | **AUC Test** |
| --- | --- | --- | --- | --- | --- |
| CSF | NfL | 152 | 1 | 0.82 | 0.85 |
|  | Ch3L1 + TNFR1 | 157 | 2 | 0.85 | 0.85 |
|  | Ch3L1 + TNFR1 + NfL | 152 | 3 | 0.91 | 0.88 |
|  | Ch3L1 + TNFR1 + sCD27 + NfL | 152 | 4 | 0.93 | 0.91 |
| Serum | osteopontin | 157 | 1 | 0.75 | 0.76 |
|  | osteopontin + MCP1 | 157 | 2 | 0.80 | 0.80 |
|  | OPN + CCL27 + MCP1 | 157 | 3 | 0.81 | 0.81 |
|  | OPN + CCL27 + IL8 + MCP1 | 157 | 4 | 0.82 | 0.82 |
| CSF/ serum combined | CSF[CD27] + serum[osteopontin] | 157 | 2 | 0.86 | 0.87 |
|  | CSF[Ch3L1 + TNFR1 + sCD27] | 157 | 3 | 0.90 | 0.89 |
|  | CSF[Ch3L1 + TNFR1 + sCD27] + serum[MCP1] | 157 | 4 | 0.94 | 0.93 |
|  | CSF[Ch3L1 + TNFR1 + sCD27] + serum[osteopontin + MCP1] | 157 | 5 | 0.95 | 0.94 |

**Supplementary Table 8 Sensitivity analysis: all biomarker concentrations were corrected for age and sex according to a linear model generated in control samples. A progression from single through combinations of multiple biomarkers to predict multiple sclerosis versus non-multiple sclerosis status.** Ch3L1: chitinase-3-like-1; MCP1: monocyte chemoattractant protein 1; NfL: neurofilament light, TNFR1: tumour necrosis factor receptor 1.

| **CSF** |  |  |  |  | **Serum** |  |  |  |
| --- | --- | --- | --- | --- | --- | --- | --- | --- |
| **Biomarkers** | **N** | **N events** | **Concordance** |  | **Biomarkers** | **N** | **N events** | **Concordance** |
| VDBP | 73 | 36 | 0.645 |  | C5 | 73 | 36 | 0.613 |
| NfL | 68 | 34 | 0.612 |  | C1inh/C1s | 67 | 31 | 0.604 |
| FB | 73 | 36 | 0.609 |  | CD27 | 73 | 36 | 0.598 |
| C1inh/C1s | 68 | 34 | 0.608 |  | TCC | 73 | 36 | 0.590 |
| C3 | 73 | 36 | 0.603 |  | FI | 73 | 36 | 0.586 |
| FI | 73 | 36 | 0.595 |  | IL18 | 73 | 36 | 0.586 |
| C5 | 73 | 36 | 0.592 |  | CXCL12 | 73 | 36 | 0.584 |
| Ch3l1 | 73 | 36 | 0.592 |  | CXCL13 | 73 | 36 | 0.581 |
| CD27 | 73 | 36 | 0.592 |  | NfL | 71 | 34 | 0.580 |
| MCP1 | 73 | 36 | 0.584 |  | C3 | 73 | 36 | 0.578 |
| CXCL12 | 73 | 36 | 0.584 |  | FH | 73 | 36 | 0.578 |
| CRP | 73 | 36 | 0.578 |  | VDBP | 73 | 36 | 0.576 |
| TCC | 73 | 36 | 0.575 |  | CCL27 | 71 | 34 | 0.576 |
| IL4 | 73 | 36 | 0.574 |  | FB | 73 | 36 | 0.575 |
| FH | 73 | 36 | 0.569 |  | OPN | 73 | 36 | 0.575 |
| TNFR1 | 73 | 36 | 0.566 |  | iC3b | 73 | 36 | 0.574 |
| C9 | 73 | 36 | 0.565 |  | CRP | 73 | 36 | 0.574 |
| IL8 | 73 | 36 | 0.562 |  | C9 | 73 | 36 | 0.572 |
| OPN | 73 | 36 | 0.561 |  | IL8 | 73 | 36 | 0.569 |
| iC3b | 73 | 36 | 0.560 |  | TNFR1 | 73 | 36 | 0.568 |
|  |  |  |  |  | Ch3l1 | 73 | 36 | 0.567 |
|  |  |  |  |  | IL4 | 73 | 36 | 0.567 |
|  |  |  |  |  | MCP1 | 73 | 36 | 0.567 |
|  |  |  |  |  | BDNF | 73 | 36 | 0.566 |

**Supplementary Table 9** **Concordance of biomarkers in predicting time to next relapse in univariate analysis (adjusted for sex and age).** BDNF: brain derived neurotrophic factor; C1inh/C1s: C1-inhibitor/C1s complex; CCL27: C-C motif chemokine ligand 27; Ch3L1: chitiase-3-like-1; CXCL: C-X-C motif chemokine ligand; IL: interleukin; MCP1: monocyte chemoattractant protein 1; NfL: neurofilament light; TCC: terminal complement complex; TNFR1; tumour necrosis factor receptor 1; VDBP: vitamin D binding protein.

|  | **Category** | **Biomarkers** | **N** | **Markers** | **Events** | **Concordance** |
| --- | --- | --- | --- | --- | --- | --- |
| Time to next relapse | CSF | VDBP | 73 | 1 | 36 | 0.65 |
|  |  | VDBP + IL8 | 73 | 2 | 36 | 0.67 |
|  |  | VDBP + CXCL12 + C1inh/C1s | 73 | 3 | 36 | 0.69 |
|  |  | VDBP + TNFR1 + chitinase-3-like-1 + C5 | 73 | 4 | 36 | 0.70 |
|  |  | VDBP + TNFR1 + chitinase-3-like-1 + C5 + CXCL12 | 73 | 5 | 36 | 0.71 |
|  |  | VDBP + CXCL12 + Factor B + CD27 + MCP1 + NfL | 68 | 6 | 34 | 0.72 |
|  | Serum | C5 | 73 | 1 | 36 | 0.61 |
|  |  | C5 + CD27 | 73 | 2 | 36 | 0.63 |
|  |  | CD27 + VDBP + C1inh/C1s | 67 | 3 | 31 | 0.66 |
|  |  | C1inh/C1s + C3 + Factor H + CXCL12 | 67 | 4 | 31 | 0.68 |
|  |  | C1inh/C1s + C3 + Factor H + CXCL12 + CD27 | 67 | 5 | 31 | 0.70 |
|  |  | C1inh/C1s + C3 + Factor H + CXCL12 + CD27 + VDBP | 67 | 6 | 31 | 0.72 |
|  | CSF/ serum combined | CSF[VDBP] + serum[C1inh/C1s] | 67 | 2 | 31 | 0.69 |
|  |  | CSF[VDBP] + serum[C1inh/C1s + Factor B] | 68 | 3 | 34 | 0.73 |
|  |  | CSF[VDBP + C1inh/C1s] + serum[Factor B + C1inh/C1s] | 63 | 4 | 29 | 0.76 |
|  |  | CSF[VDBP + C1inh/C1s] + serum[Factor B + IL4 + C1inh/C1s] | 63 | 5 | 29 | 0.79 |
|  |  | CSF[VDBP + Factor I + C1inh/C1s] + serum[Factor B + IL4 + C1inh/C1s] | 63 | 6 | 29 | 0.80 |
| Time to EDSS 6 | CSF | NfL | 68 | 1 | 17 | 0.92 |
|  |  | NfL + TNFR1 | 68 | 2 | 17 | 0.92 |
|  |  | NfL + TNFR1 + MCP1 | 68 | 3 | 17 | 0.92 |
|  |  | NfL + TNFR1 + iC3b + CXCL12 | 68 | 4 | 17 | 0.93 |
|  | Serum | C1inh/C1s | 67 | 1 | 16 | 0.90 |
|  |  | C1inh/C1s + CCL27 | 65 | 2 | 15 | 0.91 |
|  |  | C1inh/C1s + CCL27 + Factor I | 65 | 3 | 15 | 0.93 |
|  |  | C1inh/C1s + CCL27 + Factor I + osteopontin | 65 | 4 | 15 | 0.94 |
|  | CSF/ serum combined | CSF[ NfL ]+ serum[CCL27] | 66 | 2 | 16 | 0.95 |
|  |  | CSF[ NfL ] + serum[CCL27 + MCP1] | 66 | 3 | 16 | 0.96 |
|  |  | CSF[ NfL ] + serum[CCL27 + MCP1 + C1inh/C1s] | 61 | 4 | 14 | 0.97 |
|  |  | CSF[TNFR1 + C1inh/C1s] + serum[C5 + CCL27 + C1inh/C1s] | 61 | 5 | 14 | 0.97 |
|  |  | CSF[C9 + NfL ] + serum[Ch3L11 + CCL27 + VDBP + C1inh/C1s] | 61 | 6 | 14 | 0.98 |

**Supplementary Table 10** **A progression from single through combinations of multiple biomarkers to predict time to relapse and time to disability, adjusted for age and sex.** C1inh/C1s: C1-inhibitor/C1s complex; CCL27: C-C motif chemokine ligand 27; Ch3L1: chitiase-3-like-1; CXCL: C-X-C motif chemokine ligand; IL: interleukin; NfL: neurofilament light; TNFR1; tumour necrosis factor receptor 1; VDBP: vitamin D binding protein

| **CSF** |  |  |  |  | **Serum** |  |  |  |
| --- | --- | --- | --- | --- | --- | --- | --- | --- |
| **Biomarkers** | **N** | **Nevents** | **Concordance** |  | **Biomarkers** | **N** | **Nevents** | **Concordance** |
| NfL | 68 | 17 | 0.916 |  | C1inh/C1s | 67 | 16 | 0.895 |
| C1inh/C1s | 68 | 17 | 0.908 |  | CD27 | 73 | 19 | 0.865 |
| TNFR1 | 73 | 19 | 0.865 |  | CCL27 | 71 | 18 | 0.864 |
| CXCL12 | 73 | 19 | 0.861 |  | IL8 | 73 | 19 | 0.86 |
| C9 | 73 | 19 | 0.86 |  | NfL | 71 | 18 | 0.86 |
| IL4 | 73 | 19 | 0.858 |  | IL18 | 73 | 19 | 0.857 |
| C5 | 73 | 19 | 0.856 |  | Factor B | 73 | 19 | 0.856 |
| TCC | 73 | 19 | 0.854 |  | CXCL13 | 73 | 19 | 0.856 |
| Factor I | 73 | 19 | 0.853 |  | C9 | 73 | 19 | 0.855 |
| CRP | 73 | 19 | 0.853 |  | BDNF | 73 | 19 | 0.855 |
| Factor B | 73 | 19 | 0.852 |  | TNFR1 | 73 | 19 | 0.855 |
| Osteopontin | 73 | 19 | 0.852 |  | TCC | 73 | 19 | 0.854 |
| VDBP | 73 | 19 | 0.852 |  | C5 | 73 | 19 | 0.854 |
| CD27 | 73 | 19 | 0.851 |  | CXCL12 | 73 | 19 | 0.854 |
| IL8 | 73 | 19 | 0.851 |  | Ch3l1 | 73 | 19 | 0.853 |
| MCP1 | 73 | 19 | 0.851 |  | Osteopontin | 73 | 19 | 0.853 |
| C3 | 73 | 19 | 0.85 |  | IL4 | 73 | 19 | 0.853 |
| Ch3l1 | 73 | 19 | 0.85 |  | MCP1 | 73 | 19 | 0.853 |
| iC3b | 73 | 19 | 0.85 |  | C3 | 73 | 19 | 0.852 |
| Factor H | 73 | 19 | 0.849 |  | CRP | 73 | 19 | 0.852 |
|  |  |  |  |  | iC3b | 73 | 19 | 0.851 |
|  |  |  |  |  | Factor H | 73 | 19 | 0.851 |
|  |  |  |  |  | VDBP | 73 | 19 | 0.851 |
|  |  |  |  |  | Factor I | 73 | 19 | 0.85 |

**Supplementary Table 11 Concordance of biomarkers in predicting time to EDSS 6 in univariate analysis (adjusted for sex, age and disease modifying therapy).** BDNF: brain derived neurotrophic factor; C1inh/C1s: C1-inhibitor/C1s complex; CCL27: C-C motif chemokine ligand 27; Ch3L1: chitiase-3-like-1; CRP: C reactive protein; CXCL: C-X-C motif chemokine ligand; IL: interleukin; MCP1: monocyte chemoattractant protein 1; NfL: neurofilament light; TCC: terminal complement complex; TNFR1; tumour necrosis factor receptor 1; VDBP: vitamin D binding protein.

Supplementary Figure 1


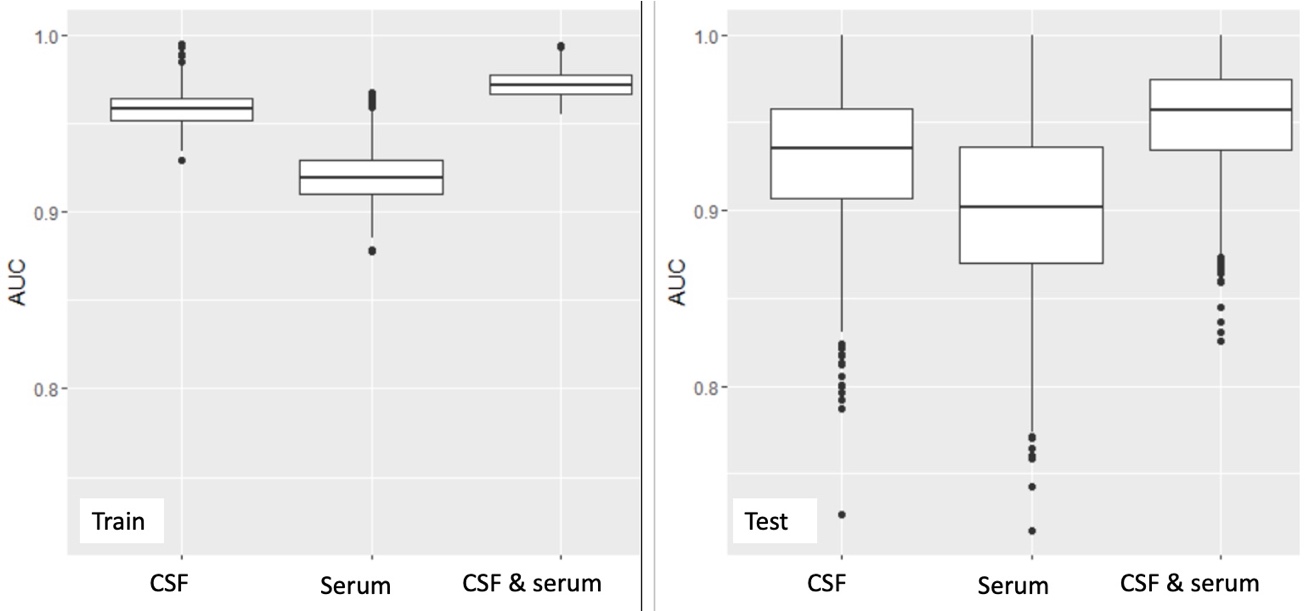


The plots show the 3 models below, run on 1000 random selection of (approximately 75%) Train and (approx. 25%) Test data. The left hand plot is the range of AUC’s from the Train data when used also as Test and the righthand plot shows the range of AUC’s when Test data is used as test.

CSF - Sex + Age + C9 + Ch3L1 + TNFR1 + CD27 + TCC

Serum - Sex + Age + iC3b + CCL27 + Osteopontin + MCP1

Both - Sex + Age + CSF[Ch3L1 + TNFR1 + CD27] + serum[Osteopontin + MCP1]
